# Complete chloroplast genome of an Endangered mangrove plant *Hernandia nymphiifolia* (C. Presl) Kubitzki (Hernandiaceae)

**DOI:** 10.1080/23802359.2018.1437830

**Published:** 2018-02-12

**Authors:** Ying Zhang, Jing-Wen Zhang, Ying-Hong Jin, Dong-Lin Li, Xiao-Ping Diao

**Affiliations:** Ministry of Education Key Laboratory for Ecology of Tropical Islands, College of Life Sciences, Hainan Normal University, Haikou, China

**Keywords:** Chloroplast genome, illumina sequencing, *Hernandia nymphiifolia* (C. Presl) Kubitzki, phylogenetic anlaysis

## Abstract

This study presents the chloroplast genome of *Hernandia nymphiifolia* (C. Presl) Kubitzki (Hernandiaceae) one Endangered mangrove species in China, which was assembled and analyzed by *de novo* assembly using whole chloroplast genome sequencing data. The accessing ID of reference sequence was MG838431. The size of the complete chloroplast genome was 157,762 bp in length with a large single-copy region (LSC) of 86,641 bp, a small single-copy region (SSC) of 18,603 bp, and two inverted repeat regions (IRS) (26,260 bp). The GC content of *H. nymphiifolia* is 39.3%. From the chloroplast genomes, 133 genes, including 83 protein-coding genes, 42 tRNA genes, and 8 rRNA genes, were predicted. Among them, 17 genes occur in IRS, containing 6 protein-coding genes, 7 tRNA genes and 4 rRNA genes. The phylogenetic analysis with 10 eudicots species and rice as the outgroup revealed that *H. nymphiifolia* was clustered with 6 Ranunculales species.

*Hernandia nymphiifolia* (C. Presl) Kubitzki (Hernandiaceae) is a mangrove assistant species (Zhang et al. 2017). In the worldwide, it occurs throughout the tropical coastal areas, from East Africa, Madagascar, Bonin Island, and New Caledonia (Michalak et al. 2010). As an evergreen tree, it is an ornamental garden plant due to its white fleshy fruit and shield blade leaves (Wang and Chen 2013). For the strong wind control abilities, it is the pioneer species with the *Casuarina equisetifolia* which can be planted in the shield forest on Hainan Island, China (Zhang et al. 2017). But in China, it has been an Endangered mangrove species for the limited location areas, and in few numbers in the last decades due to human activities (Zhong et al. 2011). The information of chloroplast genomes has been extensively applied in understanding plant genetic diversity and conservation genetics (Zhang et al. 2016). In this paper, we characterized the complete chloroplast genome sequence of *H. nymphiifolia* to contribute for further phylogenetical and protective studies of this plant.

In the study, the sample was collected from Qiong Hai, Hainan Island, China (N18°59′–19°29′, E110°7′–110°40′), and deposited at the botany laboratory of Hainan normal University, Haikou, China. Total genomic DNA was extracted from young leaves using a DNeasy Plant Mini kit (Qiagen, Mountain View, CA) according to the manufacturer’s instructions. Genome sequencing was performed on an Illumina Hiseq X 10 platform with paired-end reads of 150 bp. In total, 5408 Mb short sequence data with Q20 was 96.03% were obtained. Low-quality reads were filtered out and the remaining high-quality reads were used to assemble the chloroplast genome in SOAPdenovo (Luo et al. 2015). Then, DOGMA program was used to annotate the genes in the chloroplast genome (Wyman et al. 2004). The circular chloroplast genome map was drawn using CpGAVAS software (Liu et al. 2012). The accession number in GenBank of *H. nymphiifolia* complete chloroplast genome is MG838431.

The chloroplast genome of *H. nymphiifolia* is 157,762 bp in length and a higher GC content of 39.3%. The conserved quadripartite structure was found with a pair of IR regions of 26,260 bp that separate an LSC region of 86,641 bp and an SSC region of 18,603 bp. A total 133 protein-coding genes were annotated composed by 83 PCRs, 42 tRNA genes, and 8 rRNA genes. Among them, 17 genes occur in IRs, containing 6 protein-coding genes, 7 tRNA genes, and 4 rRNA genes. Eleven genes containing introns were found. Among them, 10 genes contain one intron and 1 gene (*ycf3*) contains two introns.

The phylogenetic relationship was studied with 11 previously reported complete chloroplast genomes, which were downloaded for the analysis. Maximum-likelyhood (ML) phylogenetic tree of 10 Eudicots species and with rice as an outgroup are shown in Figure 1 using MAFFT (Katoh and Standley 2013). The maximum likelihood algorithm was conducted using RAxML (Stamatakis 2014) implemented in Geneious ver. 10.1 (Kearse et al. 2012). The *H. nymphiifolia* chloroplast genome provides essential and important DNA molecular data for phylogenetic and evolutionary analysis for this Endangered mangrove associates species.

**Figure 1.**
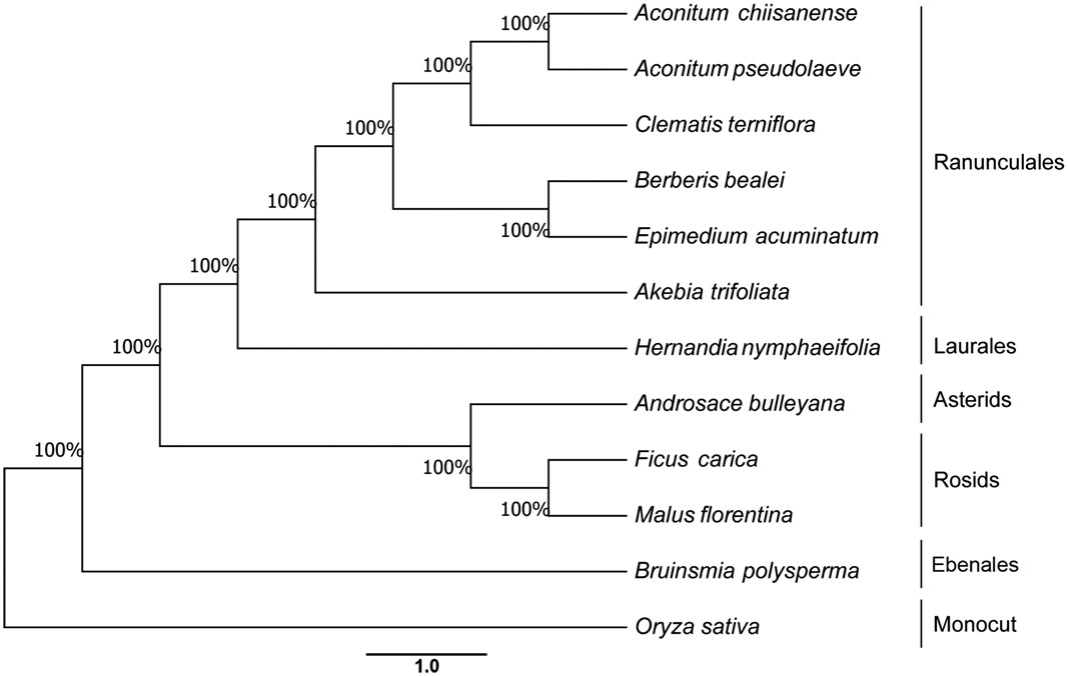
Phylogenetic relationship of the *H. nymphiifolia* chloroplast genome with 11 previously reported complete chloroplast genomes. Numbers in the nodes are the bootstrap values from 1000 replicates.
